# Association Between Anxiety and Depression and Nonalcoholic Fatty Liver Disease

**DOI:** 10.3389/fmed.2020.585618

**Published:** 2021-01-18

**Authors:** Ji Min Choi, Goh Eun Chung, Seung Joo Kang, Min-Sun Kwak, Jong In Yang, Boram Park, Jeong Yoon Yim

**Affiliations:** ^1^Department of Internal Medicine and Healthcare System Gangnam Center, Seoul National University Hospital, Seoul, South Korea; ^2^Department of Public Health Sciences, Seoul National University, Seoul, South Korea

**Keywords:** hepatic steatosis, anxiety, depression, association, mood disorder

## Abstract

**Backgrounds:** Depression and anxiety disorder are frequently seen in patients with nonalcoholic fatty liver disease (NAFLD). However, the associations between mood disorders and NAFLD have not been fully evaluated. In this study, we investigated the relationship between NAFLD and depression or anxiety in a Korean population.

**Methods:** We conducted a retrospective cross-sectional study that included subjects who underwent abdominal ultrasonography and completed a symptom questionnaire for a routine health check-up. NAFLD was diagnosed and graded according to the ultrasonography findings. Depression and anxiety were assessed using the Beck Depression Inventory and State-Trait Anxiety Inventory, respectively.

**Results:** Among the total of 25,333 subjects, the mean age was 47 years (men, 56.2%), and the prevalence rate of NAFLD was 30.9%. In the multivariate analysis, NAFLD showed a significant association with depression [adjusted odds ratio (OR) 1.43 and 95% confidence interval (CI) 1.14–1.80, *p* = 0.002] in women. Severe NAFLD significantly correlated with state anxiety and trait anxiety (adjusted OR 1.84 and 95% CI 1.01–3.37, *p* = 0.047 and adjusted OR 2.45 and 95% CI 1.08–4.85, *p* = 0.018, respectively) in women.

**Conclusions:** There was a higher tendency of women with NAFLD to suffer from depression with increase in steatosis, and severe stage of steatosis was significantly associated with anxiety in the female compared to non-NAFLD. Understanding the association between NAFLD and mood disorders may have clinical implications for reducing the prevalence of comorbidities.

## Key points

- Depression and anxiety disorder are frequently seen in patients with nonalcoholic fatty liver disease (NAFLD). However, the associations between mood disorders and NAFLD have not been fully evaluated.
- NAFLD was significantly associated with depression, and severe stage of steatosis was significantly associated with anxiety in women compared to non-NAFLD.
- These findings provide new insight in understanding the association between NAFLD and mood disorders.

## Introduction

Nonalcoholic fatty liver disease (NAFLD) is the most common cause of chronic liver disease worldwide, with increasing prevalence of up to 20–30% (1). Although NAFLD is generally a benign condition, some may progress to nonalcoholic steatohepatitis (NASH), fibrosis, and cirrhosis (2). Hepatic steatosis is commonly associated with various metabolic conditions, including cardiovascular disease (3), diabetes (4), chronic kidney disease (5), and colorectal cancer (6). In addition, NAFLD has been associated with an increased prevalence of psychological conditions such as depression and anxiety. A previous study reported that the prevalence rates of lifetime major depressive disorder (MDD) and generalized anxiety disorder were more increased in patients with NASH with more advanced histological features compared to controls (7).

Major depression is a common, recurrent disease leading to decreased quality of life, disability (8), and mortality (9). The lifetime prevalence of major depressive episode is estimated from 3 to 29.9% (10). Depression has been associated with cardiovascular diseases (11) and metabolic syndrome (12), with increased predisposition for NAFLD, suggesting shared pathogenesis of insulin resistance (13). In particular, a recent study based on US claim data has shown that depression was independently associated with NAFLD (14). In addition, generalized anxiety disorder is one of the most common anxiety disorders (15), and anxiety disorder is associated with hyperglycemia in patients with diabetes (16). This result suggests that there is a close link between anxiety and metabolic disease.

Therefore, we hypothesized that depression and anxiety disorder would be related to NAFLD and investigated the relationship between NAFLD and anxiety or depression in Korean subjects who participated in health check-ups.

## Methods

### Study Population

**Figure 1:**
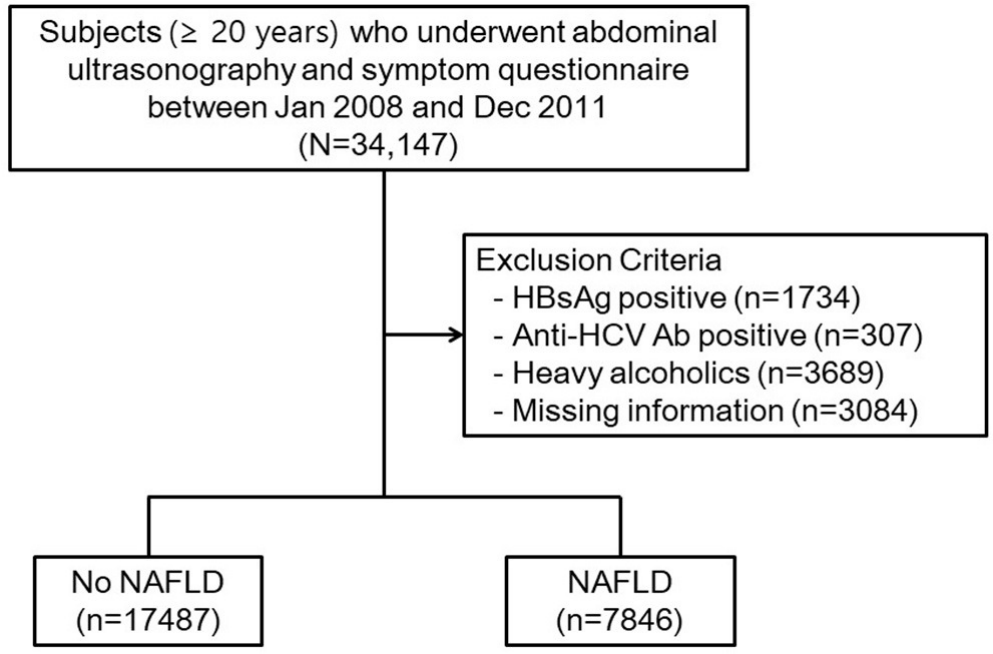
Study population. HBsAg, hepatitis B surface antigen; HCV Ab, hepatitis C virus antibody.

We performed a retrospective cross-sectional study that included subjects who underwent routine health check-up at the Seoul National University Hospital Healthcare System Gangnam Center between January 2008 and December 2011. Health examination has recently become popular in Korea because a thorough medical checkup can be performed in a few hours, and the majority of referred hospitals in Korea are now equipped with a Healthcare Center to provide such health check-ups. The subjects (age ≥ 20 years) voluntarily attended a general health check-up, while others were supported by their employers. They were mostly free of symptoms and voluntarily underwent examinations including abdominal ultrasonography and blood samplings and completed a symptom questionnaire on the same day. A schematic protocol of the study design is illustrated in Figure 1. Among the 34,147 subjects, 5,730 were excluded for potential cause of chronic liver disease, including hepatitis B virus positivity in 1,734 subjects, anti-hepatitis C virus antibody positivity in 307 subjects, and 3,689 subjects with significant alcohol consumption (>30 g/day for men and >20 g/day for women) (17). Additionally, 3,084 subjects were excluded because of missing data. The total number of eligible participants was 25,333.

The study protocol was approved by the Institutional Review Board of Seoul National University Hospital (1912–111–1089) and conformed to the ethical guidelines of the World Medical Association Declaration of Helsinki. The requirement for informed consent from individual subjects was waived because we used de-identified secondary data.

### Clinical and Biochemical Evaluations

Study data consisted of medical information based on a self-administered questionnaire and anthropometric and laboratory measurements, as described previously (18). Briefly, height and body weight were measured using a digital scale. The body mass index (BMI) was calculated as weight (kg)/height^2^ (m^2^). Waist circumference (WC) was measured by a well-trained person, at the midpoint between the lower costal margin and the iliac crest. Based on smoking status, subjects were categorized as smoker (former or current smoker) or never-smoker. Based on alcohol consumption, subjects were categorized as alcohol user or nonuser (does not drink any alcohol). Diabetes was defined as fasting glucose levels ≥126 mg/dl and/or treatment with an oral hypoglycemic agent or insulin. Systolic blood pressure and diastolic blood pressure were each measured twice, and their mean values were reported. All subjects were fasted for at least 12 h prior to blood sampling; aspartate aminotransferase (AST), alanine aminotransferase (ALT), total cholesterol, triglyceride, and high-density lipoprotein (HDL) cholesterol were measured.

### Evaluation of the State of Anxiety and Depression

The State-Trait Anxiety Inventory (STAI) scale was used to assess the level of anxiety in all subjects (19). The STAI is a well-known psychological instrument consisting of two self-report rating scales with 20 items each, for the measurement of two types of anxiety: state anxiety (how one feels at the moment, STAI-X1) and trait anxiety (how one generally feels, STAI-X2). Each item is rated between 1 and 4 depending on the frequency of target complaints (never, sometimes, often, and always), and overall scores are obtained by summing the ratings for the items (range, 20–80). We considered subjects showing moderate-to-severe state or trait anxiety by using a cut-off value of STAI-X1 ≥ 57 or STAI-X2 ≥ 59, respectively (18).

The depression status of subjects was evaluated using the Beck Depression Inventory (BDI) scale, which is one of the most commonly used self-report instruments designed to detect and measure the severity of depression in the general population (20). The BDI consists of 21 items describing symptoms of and attitudes regarding depression, and each item is rated from 0 (not at all) to 3 (extreme form of each symptom). The total score ranges from 0 to 63; the higher the score, the greater the degree of depression. By using the cutoff value of 15, subjects were classified as having no-to-mild depression (BDI < 15) or moderate-to-severe depression (BDI ≥ 15) (18).

### Diagnosis of NAFLD

Hepatic ultrasonography (Acuson Sequoia 512; Siemens, Mountain View, CA) was performed to diagnose fatty liver by experienced radiologists who were unaware of the clinical information of the subjects (21). Fatty liver was diagnosed based on characteristic ultrasonographic findings consistent with a “bright liver” and evident contrast between hepatic and renal parenchyma, focal sparing, vessel blurring, and narrowing of the lumen of the hepatic veins (22). We graded the stage of steatosis based on ultrasonographic findings: mild fatty liver as a slight diffuse increase in bright homogeneous echoes in the hepatic parenchyma and normal visualization of the diaphragm and hepatic and portal borders; moderate fatty liver as a diffuse increase in bright echoes in the hepatic parenchyma with slightly impaired appearance of intrahepatic vessels and the diaphragm; and severe fatty liver as a marked increase in bright echoes with poor or no visualization of intrahepatic vessel borders, the diaphragm, and the posterior right lobe of the liver (22).

### Statistical Analyses

Data are presented as the mean ± standard deviation for normally distributed continuous variables and as proportions for categorical variables. The Student *t* test and analysis of variance were used to analyze continuous variables, and the differences between nominal variables were compared with the chi-square test. Differences in anxiety or depression levels among the stages of steatosis were analyzed using one-way analysis of variance (ANOVA) with Tukey's honestly significant difference *post hoc* analysis. A logistic regression analysis was utilized to analyze the association of NAFLD and the stage of steatosis with depression or anxiety after adjusting for potential confounders. Among variables with a *p* value of <0.05 in univariate analyses, those with clinical importance were subjected to multivariate analyses. Statistical analyses were performed using SAS version 9.4 (SAS Institute, Cary, NC, USA) and R version 3.2.3 (The R Foundation for Statistical Computing, Vienna, Austria, http://www.Rproject.org). *p* values of <0.05 were considered statistically significant.

## Results

### Baseline Characteristics of the Study Population

Among the total of 25,333 subjects, the mean age was 47 years, and men comprised 56.2%. The prevalence rate of NAFLD was 30.9%. The subjects with NAFLD were divided into three groups based on their ultrasonographic findings of steatosis. To rule out the effects of gender, we stratified the population according to gender. The baseline characteristics of the male participants are shown in Table 1. NAFLD was more frequently observed in people who were older and smokers. Prevalence of diabetes was significantly higher in subjects with NAFLD. In addition, most of the anthropometric and laboratory variables (including BMI, WC, systolic or diastolic blood pressure, AST, ALT, total cholesterol, triglyceride, and HDL-cholesterol) were less metabolically favorable in subjects with NAFLD (*p* < 0.001). The prevalence rates of depression and anxiety were not significantly different in the NAFLD and control groups.

**Table 1:** Comparison of baseline characteristics according to nonalcoholic fatty liver disease in males.

	**No NAFLD** **(*N* = 8,067)**	**NAFLD** **(*N* = 6,169)**	**Mild** **(*N* = 2,449)**	**Moderate** **(*N* = 3,263)**	**Severe** **(*N* = 457)**	^*^P-value
Age (years)	47.5 ± 11.7	48.4 ± 10.1	48.9 ± 10.2	48.3 ± 9.9	46.2 ± 10.9	<0.001
Smoking, *n* (%)	5,820 (72.1)	4,758 (77.1)	1,902 (77.7)	2,512 (77.0)	344 (75.3)	<0.001
Alcohol, *n* (%)	7,081(87.8)	5,223 (84.7)	2,106 (86.0)	2,749 (84.2)	368 (80.5%)	<0.001
Diabetes, *n* %	347 (4.3)	537 (8.7)	167 (6.8)	310 (9.5)	60 (13.1)	<0.001
BMI (kg/m^2^)	23.4 ± 2.3	25.8 ± 2.6	25.1 ± 2.2	26.0 ± 2.5	27.8 ± 3.2	<0.001
WC (cm)	84.3 ± 6.6	90.7 ± 6.6	89.1 ± 5.8	91.2 ± 6.5	95.4 ± 8.5	<0.001
SBP (mmHg)	116.8± 13.1	120.7 ± 13.0	119.3 ± 12.9	121.3 ± 13.0	123.9 ± 11.9	<0.001
DBP (mmHg)	76.6 ± 10.5	79.9 ± 10.5	78.8 ± 10.3	80.3 ± 10.6	82.1 ± 10.0	<0.001
AST (IU/L)	22.4 ± 10.4	27.3 ± 13.4	24.5 ± 10.0	28.2 ± 13.1	35.8 ± 23.4	<0.001
ALT (IU/L)	23.4 ± 21.1	37.1 ± 24.2	30.2 ± 16.9	39.5 ± 24.7	56.8 ± 36.4	<0.001
Total cholesterol (mg/dL)	191.7± 32.5	200.8 ± 34.7	199.4 ± 34.0	201.3 ± 34.8	204.9 ± 37.2	<0.001
Triglyceride (mg/dL)	105.9± 61.5	155.8 ± 92.3	145.8 ± 83.6	159.4 ± 93.9	183.7± 115.4	<0.001
HDL-cholesterol(mg/dL)	52.8 ± 11.3	47.1 ± 923	48.0 ± 9.3	46.7 ± 9.3	44.9 ± 7.6	<0.001
Depression, *n* (%)	298 (3.7)	198 (3.2)	74 (3.0)	105 (3.2)	19 (4.2)	0.130
State_anxiety, *n* (%)	442 (5.5)	363 (5.9)	145 (5.9)	190 (5.8)	28 (6.1)	0.317
Trait_anxiety, *n* (%)	129 (1.6)	86 (1.4)	35 (1.4)	45 (1.4)	6 (1.3)	0.355

*Data are shown as the mean ± SD*.

*NAFLD, nonalcoholic fatty liver disease; BMI, body mass index; WC, waist circumference; SBP, systolic blood pressure; DBP, diastolic blood pressure; AST, aspartate aminotransferase; ALT, alanine aminotransferase; HDL, high-density lipid-cholesterol*.

* *Comparison of subjects with absence and presence of NAFLD*.

**Figure 2:**
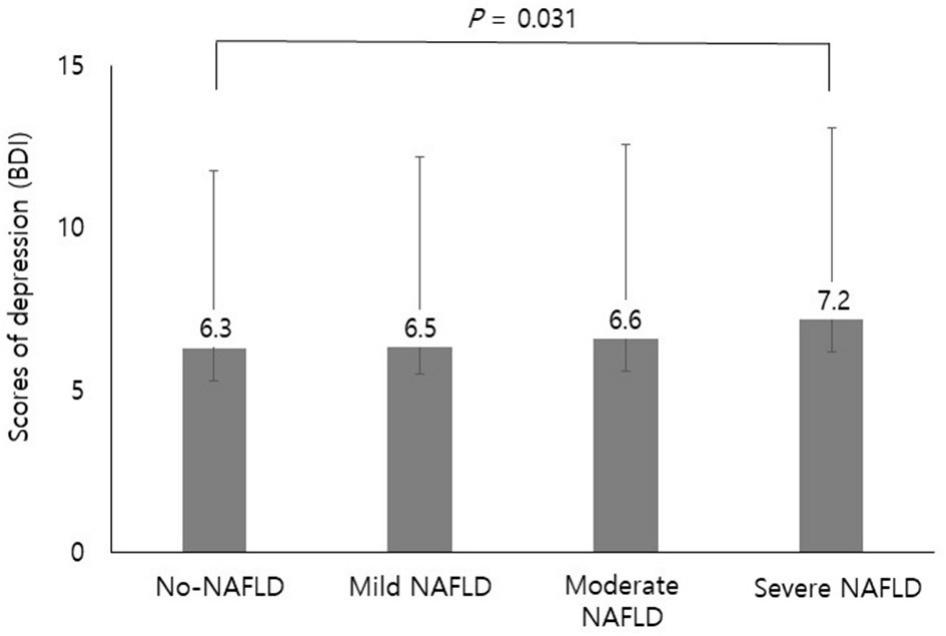
Scores of depression according to the stage of steatosis. The BDI score was significantly higher in the severe NAFLD group compared to the no-NAFLD group (7.2 ± 5.9 vs. 6.3 ± 5.5). Differences in depression levels among the stages of steatosis were analyzed using one-way ANOVA with Tukey's honestly significant difference *post hoc* analysis. BDI, Beck Depression Inventory; NAFLD, nonalcoholic fatty liver disease.

In the female group, NAFLD was more frequently observed in people who were older and never-smokers. Other baseline characteristics were almost similar to those of men except for the prevalence rates of depression which was higher in the NAFLD group compared to the control group (Table 2). Figure 2 shows the scores of depression according to the stage of steatosis. The BDI score was significantly higher in the severe NAFLD group compared to the no-NAFLD group (7.2 ± 5.9 vs. 6.3 ± 5.5, *p* = 0.031). The effect sizes of the differences were provided in the Supplementary Table 1.

**Table 2:** Comparison of baseline characteristics according to nonalcoholic fatty liver disease in females.

	**No NAFLD** **(*N* = 9420)**	**NAFLD** **(*N* = 1677)**	**Mild** **(*N* = 839)**	**Moderate** **(*N* = 759)**	**Severe** **(*N* = 79)**	^*^P-value
Age (years)	44.2 ± 10.8	53.1 ± 9.8	53.0 ± 9.7	53.2 ± 9.9	53.4 ± 9.8	<0.001
Smoking, *n* (%)	898 (9.5)	92 (5.5)	40 (4.8)	44 (5.8)	8 (10.1)	<0.001
Alcohol, *n* (%)	4,283 (45.5)	528 (31.5)	274 (32.7)	233 (30.7)	21 (26.6)	<0.001
Diabetes, *n* %	133 (1.4)	135 (8.1)	57 (6.8)	66 (8.7)	12 (15.2)	<0.001
BMI (kg/m^2^)	21.2 ± 2.4	24.9 ± 3.1	24.4 ± 2.8	25.2 ± 3.2	27.7 ± 3.1	<0.001
WC (cm)	77.7 ± 6.8	87.2 ± 7.3	86.1 ± 6.8	87.7 ± 7.3	93.5 ± 7.3	<0.001
SBP (mmHg)	108.5 ± 13.8	119.3 ± 14.3	118.9 ± 14.6	119.2 ± 13.8	124.6 ± 14.5	<0.001
DBP (mmHg)	68.1 ± 10.3	74.6 ± 10.5	74.4 ± 10.5	74.6 ± 10.3	77.5 ± 11.9	<0.001
AST (IU/L)	19.9 ± 10.5	24.8 ± 13.1	22.7 ± 10.4	26.3 ± 15.0	31.5 ± 14.6	<0.001
ALT (IU/L)	16.6 ± 17.4	27.3 ± 18.6	23.0 ± 12.2	30.6 ± 22.2	41.8 ± 22.5	<0.001
Total cholesterol (mg/dL)	190.7 ± 33.1	205.9 ± 36.1	205.2 ± 36.4	206.9 ± 35.6	204.6 ± 38.4	<0.001
Triglyceride (mg/dL)	76.5 ± 41.9	126.7 ± 70.9	118.7 ± 63.2	131.1 ± 73.7	168.8 ± 97.8	<0.001
HDL-cholesterol(mg/dL)	62.0 ± 12.7	53.2 ± 11.0	54.0 ± 11.2	52.8 ± 10.8	49.3 ± 10.2	<0.001
Depression, *n* (%)	609 (6.5)	137 (8.2)	64 (7.6)	65 (8.6)	8 (10.1)	0.012
State_anxiety, *n* (%)	1,055 (11.2)	178 (10.6)	86 (10.3)	79 (10.4)	13 (16.5)	0.509
Trait_anxiety, *n* (%)	465 (4.9)	76 (4.5)	36 (4.3)	32 (4.2)	8 (10.1)	0.518

*Data are shown as the mean ± SD*.

*NAFLD, nonalcoholic fatty liver disease; BMI, body mass index; WC, waist circumference; SBP, systolic blood pressure; DBP, diastolic blood pressure; AST, aspartate aminotransferase; ALT, alanine aminotransferase; HDL, high-density lipid-cholesterol*.

* *Comparison of subjects with absence and presence of NAFLD*.

### Risk of Depression and Anxiety With NAFLD

We investigated the relationship between depression or anxiety and NAFLD. In the univariate analysis, the presence of NAFLD had no association with depression. Regarding anxiety, the presence of NAFLD showed a significant association with state anxiety. NAFLD was associated with a 12% increase in the risk of state anxiety [odds ratio (OR) 1.12, 95% confidence interval (CI) 1.00–1.25]. When we adjusted for age, sex, diabetes, systolic and diastolic pressure, and smoking, the statistical significance has disappeared. In addition, the presence of NAFLD had no significant association with trait anxiety (Table 3).

**Table 3:** Univariate and multivariate analyses of the risk for depression or anxiety with vs. without NAFLD.

	**Univariate OR (95% CI)**	***P-*value**	**Multivariate OR (95% CI)**	***P-*value**
**Depression**				
NAFLD vs. control	1.09 (0.95–1.26)	0.205		
Mild NAFLD vs. control	1.05 (0.86–1.26)	0.642		
Mod NAFLD vs. control	1.10 (0.92–1.31)	0.274		
Severe NAFLD vs. control	1.34 (0.88–1.96)	0.147		
**State_anxiety**				
NAFLD vs. control	1.12 (1.00–1.25)	0.045	1.11 (0.99–1.24)	0.069
Mild NAFLD vs. control	1.12 (0.96–1.29)	0.151		
Mod NAFLD vs. control	1.10 (0.96–1.27)	0.172		
Severe NAFLD vs. control	1.27 (0.90–1.74)	0.155		
**Trait_anxiety**				
NAFLD vs. control	1.00 (0.83–1.21)	0.986		
Mild NAFLD vs. control	1.00 (0.77–1.28)	0.985		
Mod NAFLD vs. control	0.96 (0.74–1.23)	0.753		
Severe NAFLD vs. control	1.36 (0.75–2.26)	0.272		

*NAFLD, nonalcoholic fatty liver disease; OR, odds ratio; CI, confidence interval*.

*Multivariate analysis adjusted for age, sex, diabetes, systolic and diastolic blood pressure, and smoking*.

Next, we performed stratified analysis according to gender to exclude the influence of gender. In the univariate model, the presence of NAFLD showed a significant association with depression. NAFLD was associated with a 44% increase in the risk of depression (OR 1.44, 95% CI 1.17–1.76) in women. After adjusting for age, BMI, alcohol, diabetes, and smoking, the multivariate analysis revealed that the presence of NAFLD still showed a significant association with depression, suggesting that NAFLD has an independent association with the risk for depression (OR 1.43, 95% CI, 1.14–1.80). Although there was no statistical significance, there was a trend of increasing risk of depression according to the stage of steatosis in a dose-dependent manner (OR 1.35, 95% CI, 1.00–1.78; OR 1.52, 95% CI, 1.12–2.03; and OR 1.75, 95% CI, 0.76–3.56, mild, moderate, and severe, respectively; Table 4).

**Table 4:** Univariate and multivariate analyses of the risk for depression or anxiety in women with vs. without NAFLD.

	**Univariate OR (95% CI)**	***P-*value**	**Multivariate OR (95% CI)**	***P-*value**
**Depression**				
NAFLD vs. control	1.44 (1.17–1.76)	<0.001	1.43 (1.14–1.80)	0.002
Mild NAFLD vs. control	1.33 (1.00–1.74)	0.041	1.35 (1.00–1.78)	0.044
Mod NAFLD vs. control	1.52 (1.14–1.98)	0.003	1.52 (1.12–2.03)	0.006
Severe NAFLD vs. control	1.83 (0.81–3.60)	0.110	1.75 (0.76–3.56)	0.151
**State_anxiety**				
NAFLD vs. control	1.11 (0.93–1.32)	0.263		
Mild NAFLD vs. control	1.06 (0.83–1.34)	0.625	1.07 (0.84–1.36)	0.557
Mod NAFLD vs. control	1.08 (0.84–1.38)	0.522	1.09 (1.00–1.39)	0.478
Severe NAFLD vs. control	1.85 (0.97–3.26)	0.046	1.84 (1.01–3.37)	0.047
**Trait_anxiety**				
NAFLD vs. control	1.05 (0.80–1.35)	0.722		
Mild NAFLD vs. control	1.00 (0.68–1.39)	0.948	1.00 (0.69~1.41)	0.997
Mod NAFLD vs. control	0.97 (0.66–1.39)	0.890	0.98 (0.66–1.40)	0.908
Severe NAFLD vs. control	2.50 (1.10–4.95)	0.015	2.45 (1.08–4.85)	0.018

*NAFLD, nonalcoholic fatty liver disease; OR, odds ratio; CI, confidence interval*.

*Multivariate analysis adjusted for age, body mass index, alcohol, diabetes, and smoking*.

Regarding anxiety, the presence of NAFLD had no significant association with anxiety. However, the stage of steatosis showed significant associations with both state anxiety and trait anxiety (severe NAFLD, both *p* < 0.05), and these associations remained significant after adjusting for age, BMI, smoking, alcohol, and diabetes (adjusted OR 1.84 and 95% CI 1.01–3.37, *p* = 0.047 and adjusted OR 2.45 and 95% CI 1.08–4.85, *p* = 0.018, respectively) in women. When we performed analysis in male subjects with NAFLD, the associations were not significant (data not shown).

## Discussion

In the present study, there was a trend of increasing risk of depression according to the stage of steatosis in a dose-dependent manner in women compared with the non-NAFLD group, even after adjusting for confounding factors. In addition, severe stage of steatosis was significantly associated with state and trait anxiety in women.

These findings are in agreement with previous results showing the association between NAFLD and depression. A previous study with biopsy-proven NAFLD patients showed that more severe histological steatosis and higher NAFLD activity score were found in NAFLD patients with MDD than in those without MDD (23). Another study performed in Japan showed that depression was associated with more severe hepatocyte ballooning in pathology in patients with NAFLD (24). Although these findings were strengthened based on histological diagnosis, they are limited in their small sample size. In a study based on US claim data, those with depression were 1.6- to 2.2-fold more likely to have NAFLD compared to subjects without depression (14). A recent study based on a large Korean population showed the dose-dependent pattern of the relationship between the risk for depression and the ultrasonographically graded severity of NAFLD (25). Unlike our results, the significant association was found especially in men in the previous study, and these different results may be due to the heterogeneous study population, including the mean age of the subjects (40 vs. 47 years) and the prevalence of depression (10.6 vs. 4.7%), and the different definitions regarding depression, which were based on the Center for Epidemiological Studies-Depression scale. Regarding anxiety, few studies have evaluated the association with NAFLD. A previous study based on patients with biopsy-proven NAFLD showed that anxiety tended to be associated with less hepatocyte ballooning; however, there was no significant association between anxiety and portal fibrosis (24).

Previous studies have investigated the influence of mood disorder on the therapeutic effect of liver disease. Tomeno et al. suggested that NAFLD patients with depression had poor response to standard treatment for NAFLD (23). A meta-analysis showed that psychological distress such as depression and anxiety was associated with mortality in chronic liver disease (26). Taken together, NAFLD patients with depression may experience worse clinical outcomes than those without; thus, active screening and appropriate treatment of depression may prevent the development or progression of NAFLD.

It is well known that there is gender difference in the prevalence of NAFLD and depression. While NAFLD is more common in men (1), depression is more prevalent in women (27). In our study, women had a prevalence of depression (6.7%) up to twice that of men (3.5%), whereas men had a higher prevalence of NAFLD (43.3%) than women (15.1%). Thus, we stratified the population according to gender, and the rate of individuals having both depression and NAFLD increased after adjusting for gender, resulting in a significant association between NAFLD and mood disorder only in women.

The pathogenesis underlying the association between NAFLD and depression or anxiety has not been fully elucidated. There are possible explanations for the close link between hepatic steatosis with insulin resistance and poor glycemic control, both of which have been associated with depression (28) or anxiety (16). The involvement of insulin signaling on brain mechanisms related to depression indicates that insulin resistance may be one of the main pathogenic drivers for NAFLD (29). In addition, increased inflammation markers (30) and pro-inflammatory cytokines, such as tumor necrosis factor-alpha and interleukin-6, in patients with mood disorders may offer another plausible explanation for the association of these mood disorders with NAFLD (31). Another line of evidence suggests the involvement of the serotonin pathway. Experimental murine models of NAFLD showed that serotonin played an important role in the pathogenesis of NASH. Additionally, the expression of monoamine oxidase-A, one of the main enzymes catalyzing monoamines such as serotonin, increased in patients with NASH (32).

The strengths of this study include the large sample size, which ensured the robustness of results, and the STAI scale, which was able to assess both types of anxiety, including trait or chronic anxiety, which reflects a person's permanent characteristics, and state or acute anxiety, which reflects a recent state (33).

There are several limitations in this study. First, due to its observational study design, the results need to be interpreted cautiously. The association between NAFLD and mood disorder may not imply causality. Further research with longitudinal study design is needed to clarify the causal relationship. Second, since depression or anxiety was defined based on the self-reporting questionnaire, there is the potential for over- or underreporting-related symptoms. Because the recommended cutoff points of BDI or STAI is not consistent among specific disease or populations (34–38), we used the cutoff values referring to previous study based on Korean population (18). Third, although liver biopsy is considered to be a gold standard for the diagnosis of NAFLD, it was assessed only by ultrasonography in this study. Thus, there may be a limitation of lack in accurate diagnoses for mild steatosis. And although the fibrosis was found to be a critical factor in mental health of NAFLD patients (39), we could not evaluate the fibrosis stage. In clinical practice, liver biopsy is not typically used in healthy subjects due to its invasiveness. Thus, radiographic techniques such as ultrasonography or magnetic resonance imaging are used for the diagnosis of NAFLD. Fourth, we could not assess the influence of the history of anti-depressant medications. Fifth, as a result of this study using the previous cohort (18), there is a limitation that there is no information on the history of hypertension. However, systolic and diastolic blood pressure were adjusted as variables in the multivariate analysis. Finally, because we could not exclude all patients that have drinking habit, there might be bias in the results.

In conclusion, NAFLD was significantly associated with depression in women, and severe NAFLD was significantly associated with anxiety in the female group compared to non-NAFLD. Understanding the association between NAFLD and mood disorders may have clinical implications for reducing the prevalence of comorbidities, and appropriate screening and active referrals for early treatment of depression may be suggested for patients with NAFLD.

## Data Availability Statement

The raw data supporting the conclusions of this article will be made available by the authors, without undue reservation.

## Ethics Statement

The studies involving human participants were reviewed and approved by Institutional Review Board of Seoul National University Hospital. Written informed consent for participation was not required for this study in accordance with the national legislation and the institutional requirements.

## Author Contributions

GC conceived the idea, determined the study design, collected the data, drafted and revised the manuscript. JC and SK collected the data, performed the statistical analysis and revised the manuscript. MK, JYa, and JYi collected and reviewed the data, and revised the manuscript. BP performed the statistical analysis. All authors contributed to the article and approved the submitted version.

## Conflict of Interest

The authors declare that the research was conducted in the absence of any commercial or financial relationships that could be construed as a potential conflict of interest.
