# Supplementary material for: Association Between Anxiety and Depression and Nonalcoholic Fatty Liver Disease
Source: Front Med (Lausanne). 2021 Jan 18;7:585618. doi: 10.3389/fmed.2020.585618 (PMC7848018; doi:10.3389/fmed.2020.585618)
Supplement: Supplementary file 1 [file Table_1.pdf]

Supplementary Table 1. The effect sizes of differences regarding baseline characteristics according to the presence and severity of NAFLD.

	No NAFLD	NAFLD	Mild	Moderate	Severe
Age	0.291	-0.474	-0.411	-0.495	-0.693
Smoking	1.652	2.121	2.204	2.091	1.75
Alcohol	1.004	1.279	1.293	1.288	1.286
Diabetes	0.174	0.024	0.001	0.028	-0.059
Body mass index	0.936	0.298	0.285	0.26	0.05
Waist circumference	0.985	0.504	0.47	0.508	0.239
Systolic blood pressure	0.617	0.1	0.029	0.153	-0.057
Diastolic blood pressure	0.814	0.501	0.429	0.55	0.416
AST	0.236	0.193	0.178	0.135	0.221
ALT	0.351	0.455	0.491	0.382	0.495
Total cholesterol	0.03	-0.144	-0.165	-0.157	0.007
Triglyceride	0.558	0.353	0.365	0.335	0.14
HDL-cholesterol	-0.769	-0.6	-0.577	-0.6	-0.487
Depression	-0.126	-0.215	-0.206	-0.229	-0.233
State_anxiety	-0.208	-0.173	-0.159	-0.169	-0.331
Trait_anxiety	-0.189	-0.186	-0.172	-0.173	-0.387

NAFLD, nonalcoholic fatty liver disease; AST, aspartate aminotransferase; ALT, alanine aminotransferase; HDL, high-density lipid-cholesterol

Effect size indicates the standardized mean difference.

Standardized mean differences = differences in means or proportions divided by the pooled standard deviation.

+ values favor males and - values favor females.
